# Assessing and promoting partnership between patients and health‐care professionals: Co‐construction of the CADICEE tool for patients and their relatives

**DOI:** 10.1111/hex.13253

**Published:** 2021-05-05

**Authors:** Marie‐Pascale Pomey, Nathalie Clavel, Louise Normandin, Claudio Del Grande, Djahanchah Philip Ghadiri, Isabel Fernandez‐McAuley, Antoine Boivin, Luigi Flora, Annie Janvier, Philippe Karazivan, Jean‐François Pelletier, Nicolas Fernandez, Jesseca Paquette, Vincent Dumez

**Affiliations:** ^1^ School of Public Health University of Montreal Montreal QC Canada; ^2^ Centre de recherche du Centre Hospitalier de l’ Université de Montréal Montreal QC Canada; ^3^ Centre of Excellence on Partnership with Patients and the Public Montreal QC Canada; ^4^ Ingram School of Nursing McGill University Montreal QC Canada; ^5^ Department of Management HEC Montréal Montreal QC Canada; ^6^ Department of Family Medicine University of Montreal Montreal QC Canada; ^7^ Faculté de Médecine Université Nice Sophia Antipolis Nice France; ^8^ Department of Pediatrics University of Montreal Montreal QC Canada; ^9^ Department of Psychiatry and Addictology University of Montreal Montreal QC Canada; ^10^ Department of Education Université du Québec à Montréal Montreal QC Canada; ^11^ Faculty of Medicine University of Montreal Montreal QC Canada

**Keywords:** clinical care, co‐construction, health‐care surveys, patient involvement, patient partnership, tool

## Abstract

**Context:**

Partnership between patients and health‐care professionals (HCPs) is a concept that needs a valid, practical measure to facilitate its use by patients and HCPs.

**Objective:**

To co‐construct a tool for measuring the degree of partnership between patients and HCPs.

**Design:**

The CADICEE tool was developed in four steps: (1) generate key dimensions of patient partnership in clinical care; (2) co‐construct the tool; (3) assess face and content validity from patients’ and HCPs’ viewpoints; and (4) assess the usability of the tool and explore its measurement performance.

**Results:**

The CADICEE tool comprises 24 items under 7 dimensions: 1) relationship of **C**onfidence or trust between the patient and the HCPs; 2) patient **A**utonomy; 3) patient participation in **D**ecisions related to care; 4) shared **I**nformation on patient health status or care; 5) patient personal **C**ontext; 6) **E**mpathy; and 7) recognition of **E**xpertise.

Assessment of the tool's usability and measurement performance showed, in a convenience sample of 246 patients and relatives, high face validity, acceptability and relevance for both patients and HCPs, as well as good construct validity.

**Conclusions:**

The CADICEE tool is developed in co‐construction with patients to evaluate the degree of partnership in care desired by patients in their relationship with HCPs. The tool can be used in various clinical contexts and in different health‐care settings.

**Patient or Public Contribution:**

Patients were involved in determining the importance of constructing this questionnaire. They co‐constructed it, pre‐tested it and were part of the entire questionnaire development process. Three patients participated in the writing of the article.

## 1 INTRODUCTION

In recent years, patient engagement in direct care has received more attention in health care and is being implemented internationally.^1^, ^2^, ^3^ Partnership in care takes patient engagement further, by considering patients as real partners in the care process and as full‐fledged members of the health‐care team.^4^ This innovative approach to care is built on different approaches to care, including patient‐centred care, patient empowerment and shared decision‐making, but goes further by recognizing: (1) the experiential knowledge they have gained from living with a disease and from experiencing health‐care services; (2) patients as full‐fledged care team members, like the health‐care professionals (HCPs); and (3) patients as capable of making the most appropriate decisions for themselves.^5^ For many patients, their doctors’ understanding of their disease is inconsistent with their experience with that disease.^5^ Because patients understand the realities associated with their condition and the impacts of the disease and its treatment on their lives, their perspective enhances HCPs’ expertise and should be considered in all care decisions.^4^, ^6^, ^7^, ^8^

Patient partnership of care refers to the co‐construction between health professionals and patients to assess the patients’ participation in the decisions made regarding their care, and co‐build intervention to support and accompany patients as they become increasingly autonomous in their decision‐making and ability to influence their health.^4^, ^9^ However, patients differ in their ability to be autonomous, and their situation may change in the course of their care trajectory. Health professionals do not currently have the tools to fully perceive this reality and act based on patients’ real needs.

Recently published frameworks for evaluating different aspects of patient engagement have underscored the importance of evaluating patients’ involvement, not only at the organizational level, but also at the clinical level.^10^, ^11^, ^12^, ^13^ A tool has been developed to measure patients’ contributions and public involvement at the organizational level,^14^ thereby supporting the quality of public and patient engagement in health service organizations. The Public and Patient Engagement Evaluation Tool (PPEET) makes it possible to concurrently consider the points of view of members of the public/patients/family members, managers and board members/senior managers. However, the tools currently available at the clinical level cover only some of the aspects of this partnership as it is perceived by patients and health‐care professionals.^8^, ^15^, ^16^, ^17^, ^18^ A review of the tools carried out when this study began and a complementary scoping review carried out in 2021^19^ show four limitations in recent tools developed and used to assess patient engagement at the clinical level. First, most of the tools evaluate dimensions related to one or two different concepts of patient engagement (patient‐centeredness or empowerment or shared decision‐making).^20^, ^21^, ^22^, ^23^, ^24^, ^25^, ^26^, ^27^, ^28^, ^29^, ^30^, ^31^ Accordingly, no tool offers an exhaustive assessment of all the core concepts and dimensions on which the new partnership in care approach is built. Second, tools assessing preferences of patient engagement in care are scarce, and almost all tools evaluate the patient engagement experience, while measuring engagement should focus on both the patient's preferences and experience for engagement or the HCPs’ perspective. Third, the scoping review showed that no tools were developed in co‐construction with patients from development to validation. Finally, very few tools were generic and thus developed to be used in different contexts of care, in inpatient or outpatient clinical settings, with patients exhibiting various chronic or acute diseases. These results led us to realize that this emerging concept requires a valid, practical instrument that can be used by patients and HCPs at the clinical level.

This article is therefore intended to bridge the gap in assessments of patient partnership at the clinical level by sharing the results of a three‐year collaboration between patients, HCPs and researchers to develop a tool that could be used by a wide range of organizations in the health system. This tool, called CADICEE, is intended as a means to help patients and HCPs discuss the basis for the partnership relationship and identify potential partnership gaps. More broadly, it could also help create a database on how patients perceive the extent of their partnership with their HCPs.

This research project received ethics approval from the research ethics committee (CER‐2017‐018 and 18.045) of the Centre de recherche du Centre hospitalier de l’Université de Montréal (CRCHUM).

## 2 METHODS AND FINDINGS

The CADICEE tool was developed in four steps, in accordance with the Guidance for Reporting Involvement of Patients and the Public Long Form (GRIPP2‐LF),^32^ to guarantee the quality and reporting of patient involvement:

**Step 1:** Generation of the core dimensions of patient partnership in clinical care, based on a qualitative approach that was, in turn, based on the clinical sociology method^33^, ^34^, ^35^;
**Step 2**: Co‐construction of the tool, based on the dimensions that emerged in Step 1;
**Step 3**: Assessment of face and content validity, from the patients’ and HCPs’ viewpoints; and
**Step 4**: Assessment of the tool's usability and exploration of its measurement performance.

### 2.1 Step 1: Generating the core dimensions of partnership in clinical care

#### 2.1.1 Methods

To be able to co‐construct the tool, we decided to appeal to patients who understand the patient partnership context. We seek to hire expert patients on that topic regardless of their degree of literacy. To do so, we asked the person in charge of the bank of patients participating in courses for future health‐care professionals at the Université de Montréal on care partnerships.^36^, ^37^, ^38^ From this bank, we targeted those who: (1) had at least one chronic disease, and (2) were capable of talking to students about their experiences with the disease and how they mobilize their experiential knowledge to become full partners in the care team, so that the team's decisions would be based on their life plan.^4^, ^6^, ^37^

Drawing on the clinical sociology method,^33^, ^34^, ^35^ patients selected were asked to write a three‐ to five‐page account of their care experience in the context of a single consultation with an HCP. A qualitative thematic analysis was performed by the research team, composed of patient‐researchers (n = 3) and researchers (n = 8), to analyse the accounts of these patients and highlight the various dimensions covered.^39^ Each text was analysed using the NVivo (version 10) software. Two techniques were used in the coding: primary open coding, followed by thematic and selective coding,^40^, ^41^ as per the grounded theory approach.^42^

#### 2.1.2 Findings

Of the 243 patients involved in care partnership courses, 85 were approached, 34 responded, and following interviews, 15 were retained with various characteristics. The 15 expert patients were mostly women (13/15; 87%), over 45 years old (10/15; 66%) (see Table 1) and with a variety of chronic diseases (including cancer, metabolic diseases, degenerative diseases and autoimmune diseases).

**TABLE 1:** Sociodemographic characteristics of the patients who participated in the development of the tool (steps 1 and 2)

Characteristics of expert patients	N = 15 patients, Number (%)
Age	
26‐35	3 (20.0)
36‐45	2 (13.3)
46‐55	6 (40.0)
56‐65	2 (13.3)
66‐75	2 (13.3)
Gender	
Female	13 (86.7)
Male	2 (13.3)
Type of expert patient	
Patient	13 (86.7)
Member of patient's family	2 (13.3)
Language	
French	14 (93.3)
English	1 (6.7)

Development followed an iterative process in which the expert patients were asked to participate in the entire process of co‐constructing the tool as a patient research advisory group that would consider the literature review, patient accounts and the consensus obtained in face‐to‐face meetings. The analysis of the patients’ accounts and the literature review were presented to the patients' advisory group in September 2015. Three rounds of deliberation were used to reach a consensus on the core dimensions of partnership in care: during each round, participants were asked to rate each dimension on a 10‐point scale, ranging from 1 (unimportant) to 10 (very important). In each round, patients were invited to provide justifications for their ratings, refine the wordings and propose theme definitions. At each meeting, we held discussions to review overall scores and participants made comments about the proposed theme definitions. All participants attended each round. At the end of the three rounds, the group reached a consensus on each of the seven dimensions that had emerged^43^: **C**onfidence/trust, **A**utonomy, participation in **D**ecision‐making, sharing **I**nformation, personal **C**ontext, **E**mpathy and **E**xpertise, hence the acronym CADICEE for the tool (see Table 2).

**TABLE 2:** Dimensions and items of the CADICEE tool

Dimension of the CADICEE tool	Item
**C**onfidence/Trust	Did you need to feel mutual confidence / trust with this professional? Did you feel that you could have confidence in / trust the professional consulted? Did you feel that this professional had confidence in / trusted you? Did you think you were open to this professional's point of view? Were you open to this professional's point of view?
**A**utonomy	Did you want this professional to help you become more autonomous / independent? Did this professional help you become more autonomous / independent (through advice, opinion, etc)?
(Participation in) **D**ecision‐making	Did you feel a need to participate in decisions related to your care? Did you participate in the decisions related to your care?
(Sharing) **I**nformation	Did you need important information about your health or care from this professional? Did you receive important information about your health or care? Did you receive important information that you did not want? Did you need to give important information about your health or care to this professional? Did you give important information on your health or your care?
Personal **C**ontext	Did you need your care to be adjusted to your personal context? Was your care adapted to your personal context?
**E**mpathy	Did you need to feel empathy from this professional? Did you feel empathy from this professional? On your part, did you show empathy towards this professional?
**E**xpertise	Did you think you had useful expertise for this consultation? Did you use your expertise in what was said or done? Did you need your expertise to be recognized and taken into account by professional? Did you feel that this professional recognized your expertise and took it into account? Did you think that this professional had useful expertise for this consultation? Did this professional show he had useful expertise based on what was said and done?

For our expert patients, partnership in care emerges in relation to patients’ needs in the context of specific consultations; it is not an unconditional attribute of care. This approach to partnership in care shaped the entire structure of the CADICEE tool. The assessment of each partnership dimension and its related items was designed to capture the needs of patients regarding their interactions with one HCP and their perceptions of the quality of their interactions with the HCP during the consultation, making it possible to assess discrepancies between patient needs and experiences of care, which could then serve as indicators to build awareness among patients and HCPs of their relational issues and improve their partnership over time.

### 2.2 Step 2: Co‐construction of the tool, based on the dimensions from Step 1

#### 2.2.1 Methods

After stabilizing the seven core dimensions of partnership in care, patients and researchers met to discuss the tool's structure and the questions to be asked, and determine measurement scales. In addition, to ensure that the wording used in the questionnaire would be accessible to everyone who may potentially use it, a literacy expert was consulted to ensure that people with a Grade 6 level of literacy (children between 11 and 12 years old) could understand the tool, based on the Simple Measure of Gobbledygook (SMOG) readability formula.^44^, ^45^ The SMOG formula is based on the number of words containing three or more syllables and has been found exceptionally effective in the health‐care setting.

#### 2.2.2 Findings

The research team and the patient research advisory group exchanged emails and met twice to draft a preliminary version of the tool, which consisted of 26 questions grouped in two parts: Part 1 consisted of 11 questions on patients’ needs prior to the consultation based on the seven dimensions of the tool; and Part 2 gathered information on patients’ perceptions of their experience during the consultation based on the same dimensions (13 questions). Additionally, questions designed to identify participants’ sociodemographic characteristics, the main reason for the consultation, the presence of a chronic disease and HCPs frequently consulted were introduced.

To rate the items, the researchers initially proposed a different measurement scale for patients’ needs and care relationship experiences. To assess needs (Part 1), a unipolar 6‐point scale was proposed, ranging from 0 (not at all) to 5 (very much). Care relationship experiences (Part 2) were assessed using a bipolar 11‐point scale ranging from −5 (extremely negative label tailored to each item) to 5 (extremely positive label). Both scales included a ‘Don't know/Not applicable’ option.

### 2.3 Step 3: Assessment of face and content validity from patients’ and HCPs’ viewpoints

#### 2.3.1 Methods

The tool was sent to two different types of participants. One group consisted of new patient partners not previously involved in the project who were also teaching health professionals. Patients selected had to meet two criteria: (1) be a patient‐as‐trainer taking part in partnership courses given to students in the health sciences and psychosocial sciences; and (2) be a patient or caregiver having regular contact with health‐care providers. The second group consisted of HCPs who had helped teach patient partnership to future HCPs.^6^, ^37^ A written invitation was sent to teachers who had participated in these courses for over three years (n = 23).

Participants from the two groups received the tool and an anonymous evaluation grid assessing their understanding of the objective/purpose of the tool, its overall readability, the appropriateness of the measured dimensions and the individual items (wording, relevance), as well as its ease of use and the appropriateness of the measurement scales. For each question in the evaluation grid, participants were asked to check a dichotomous Yes/No box and were encouraged to add a comment justifying their answer.

#### 2.3.2 Findings

A 14 patients were available to participate in reviewing the tool. Despite the wish to have a highly diverse group, these patients were mostly women (10/14; 71%) and over 45 years old (8/14; 57%). Patients who tested the tool had a variety of chronic diseases (including cancer, metabolic diseases, degenerative diseases, autoimmune diseases, respiratory and genetic diseases) and sociodemographic characteristics (see Table 3).

**TABLE 3:** Sociodemographic characteristics of the patients who participated in the assessment of the face and content validity of the tool (step 3)

Characteristics of expert patients	n = 14 patients, Number (%)
Age	
26‐35	1 (7.1)
36‐45	5 (35.7)
46‐55	2 (14.3)
56‐65	4 (28.6)
66‐75	2 (14.3)
Gender	
Female	10 (71.4)
Male	4 (28.6)
Type of expert patient	
Patient	13 (92.9)
Member of patient's family	1 (7.1)
Language	
French	13 (92.9)
English	1 (7.1)

Among the 23 HCPs, 10 HCPs responded to the invitation. They represented various health professions (managers (n = 2), physicians (n = 2), pharmacists (n = 1), nurses (n = 2), a dietician (n = 1), social workers (n = 1) and a physiologist (n = 1)).

The research team synthesized the evaluation grids from both patients and HCPs. All of the patients and 90% of the HCPs found the tool relevant and useful. The HCPs mentioned that the tool led them to think about care relationship dimensions that they had not thought about before, such as patients demonstrating empathy for HCPs. In addition, all of the patients and 80% of the HCPs found the tool straightforward. Almost all of the HCPs (90%) were comfortable using the two measurement scales of the tool, whereas 71% of the patients were comfortable with these scales.

In a face‐to‐face meeting, the research team and the patient research advisory group discussed the comments. Based on a consensus reached among patients and researchers, the team decided to revise some items in the tool and the measurement scales: (1) to record the HCP’s gender, in the first part of the tool; (2) to specify the relevance of the information provided, in the shared information aspect; (3) to document whether the patient received a prescription (or a renewal) during the consultation; (4) to allow the patient to decide whether and how the data should be shared with the HCP; and (6) to measure how much the consultation met the patient's needs overall.

To improve the usability and acceptability of the measurement scales, it was agreed that both needs and experiences of care should be measured on an identical scale with less negative value labels. Therefore, a 4‐point unipolar scale measuring the level of extent (from not at all to very much) was proposed. To avoid redundancy and reduce the time required to complete the tool, the first and second parts were merged by grouping items related to relationship needs and experiences for each dimension. The final version of the CADICEE tool consists of 27 questions: general appreciation of the consultation (n = 1); partnership needs (n = 11); partnership experiences (n = 13); importance of the different dimensions (n = 1); and how the answers should be used (n = 1). In addition, general information questions (n = 12) are asked.

The questionnaire was first written in French and then translated into English, following the methodology proposed by the Agency for Healthcare Research and Quality ^46^ and the methodology recommended by the United States Bureau of the Census.^47^

### 2.4 Step 4: Assessment of the tool's usability and measurement performance

#### 2.4.1 Methods

The final step consisted of testing the feasibility of using the tool in different clinical environments (primary care, mental health, rehabilitation, oncology, general hospitalization) and exploring its measurement performance. A letter was sent to three health and social service institutions (two in urban settings and one in a rural setting), and two French‐run primary care clinics. The objective of the phase was to test the tool's usability: how to administer it (paper versus electronic tablet), highlight clinical contexts that were favourable or not to distributing the tool, and validate that the tool can be completed for different types of HCPs. It was also possible to interview French‐speaking patients and relatives about the tool's structure, layout, comprehensibility (wording) and the interest in completing it, the time taken to complete it, patients’ perceptions of its usefulness in promoting and evaluating their partnership, and whether they were willing to share the results of the questionnaire with their professionals.

During this step, we also explored the measurement performance of the CADICEE tool. Use of the categories on the response scale, response distributions and proportion of missing values were assessed, overall and per item. Non‐parametric tests (due to the ordinal nature of the data) were conducted to measure the correlation between CADICEE items and investigate differences in response patterns among relevant patient subgroups. Lastly, we checked whether the number and severity of negative discrepancies reported by respondents between needs and experience of care in CADICEE dimensions would be associated with their general appreciation of the consultation. Statistical analyses were conducted in SPSS version 26 (IBM Corp.), and the significance level was set at α < 0.050. Given the nature of the tool, internal consistency and factorial validity were not deemed appropriate criteria to examine.^48^, ^49^ CADICEE items refer to distinct aspects of care partnerships, and any combination of responses could be consistent with a particular experience of care. There is no expectation that items should be correlated (or not) with one another, even within the same dimension (eg lack of correlation between relevant information received from and communicated to the HCP is completely plausible and would not reflect a measurement problem with the ‘Shared information’ dimension).

#### 2.4.2 Findings

The three health and social service institutions and the two primary care clinics volunteered to test the tool in practice. In total, 262 patients in various clinical settings tested the tool, of which 246 completed it (94% completion rate): 116 in primary care, 42 in oncology, 41 in mental health, 12 in rehabilitation and 35 as patient advisors. The sample was diversified in terms of gender (56% women) and covered a wide range of age groups, education levels and life experience with a chronic disease. 67% (n = 164) completed the questionnaire regarding a consultation with a doctor (see Table 4).

**TABLE 4:** Characteristics of patients who have tested the CADICEE tool (step 4)

	N = 246 (%)^a^
Clinical setting	
Primary care	116 (47.2)
Oncology	42 (17.1)
Mental health	41 (16.7)
Rehabilitation	12 (4.9)
Patient advisors	35 (14.2)
Respondent status	
Patient	206 (83.7)
Relative (present during the consultation)	38 (15.4)
Gender	
Female	138 (56.1)
Male	103 (41.9)
Age group	
18‐44 y	83 (33.7)
45‐64 y	96 (39.0)
65+	62 (25.2)
Education level	
Elementary	16 (6.5)
High school	59 (24.0)
College / vocational	57 (23.2)
University	106 (43.1)
Had at least one chronic disease	
Yes	147 (59.8)
Since <1 y	19 (7.7)
Since 1‐5 y	36 (14.6)
Since 6‐10 y	28 (11.4)
Since 11‐20 y	30 (12.2)
Since >20 y	27 (11.0)
No	95 (38.6)
Health‐care professional consulted	
Doctor	164 (66.7)
Nurse	41 (16.7)
Psychologist	17 (6.9)
Other	22 (8.9)

^a^ Percentages in each cell may not add up to 100% due to missing values.

The overall recruitment rate was 60% (262 out of 438 patients contacted), although rates were lower in specialized mental health and rehabilitation services (51% and 47%, respectively). All patients coming from settings, other than mental health and rehabilitation services, indicated that the tool was user‐friendly and that the questions were clear and were not too time‐consuming to complete (13 minutes on average). The ‘extent’ scale was easy to use. The definitions given for each dimension were perceived as essential to a clear understanding of the context of the questions.

More than 70% of respondents indicated that they were willing to share the results from their questionnaire with their HCP to disclose their expectations and see how they could work with their HCP to improve the quality of their relationship. Another 17% agreed to share their responses only if they were compiled with those of other patients as a statistical summary. More patients in mental health and rehabilitation services had reservations about allowing their HCPs to see the results (33% and 18%, compared with 11% in primary care and oncology and 0% as patient advisors).

Regarding measurement performance of the tool, the full range of the 4‐point ‘extent’ response scale was used on all CADICEE items except one (no one reported perceiving, before the consultation, that the professional had no useful expertise at all). Range and mean use of response categories across the 24 CADICEE items are reported in Table 5. The extreme response categories (1 = not at all and 4 = very much) were, on average, significantly used more often for items assessing needs compared with items assessing experience of care, while the opposite was observed for the other two response categories (2 = very little and 3 = moderately). Medians varied between 3 (18 items) and 4 (6 items), and interquartile range (IQR) varied between 1 (21 items) and 3 (1 item). The ‘Don't know/Not applicable’ response category was seldom used by respondents (mean use per item = 1.8%, SD = 1.5), and missing values were found ranging from 0% to 6.1% (mean = 2.8%, SD = 1.5).

**TABLE 5:** Summary use of the 4‐point response categories across CADICEE items, in percentages

Response categories	Range across all 24 items: min‐max	Overall mean use per item (SD)	Mean use (SD) for items assessing needs vs experience of care, respectively	*P* *
(1) Not at all	0%‐24.4%	4.7% (4.6)	6.5% (6.3) vs 3.3% (1.5)	.037
(2) Very little	2.4%‐35.8%	11.6% (7.4)	8.2% (4.6) vs 14.5% (8.2)	.039
(3) Moderately	21.5%‐52.4%	39.4% (9.4)	33.6% (9.3) vs 44.3% (6.3)	.004
(4) Very much	21.5%‐70.3%	39.7% (13.9)	47.4% (15.5) vs 33.1% (8.3)	.016

* Mann‐Whitney *U* test, two‐tailed exact *P*‐values.

Spearman's rank‐order correlation (*r*_s_) matrices were separately produced for the 11 CADICEE items assessing needs and the 13 items assessing experience of care. For items assessing needs, 27 of 55 unique bivariate combinations (n*[n‐1]/2, where n is number of items) were statistically significant. The highest correlations observed were between ‘Did you think you had useful expertise for this consultation?’ and ‘Did you need your expertise to be recognized and taken into account by the professional?’ (*r*_s_ = .44, *P* < .001), and between ‘Did you need important information about your health or care from this professional?’ and ‘Did you need to give important information about your health or care to this professional?’ (*r*_s_ = .43, *P* < .001), which is not surprising. Most of the remaining significant coefficients (19/25) were indicative of ‘low’ correlation (<.30),^50^ which suggests that respondents understood the distinct character of CADICEE dimensions measured in the tool. Significant and stronger correlations were more frequent between items assessing experience of care. Twenty‐one of 78 unique *r*_s_ coefficients were >.40, although only three were in the moderate or strong range (.50‐.75),^50^ which concerned items about Confidence/trust and Empathy, which are related concepts.

We also conducted median tests to detect differences in the position and shape of response distributions for each CADICEE item depending on clinical setting, HCP consulted, or respondents’ age, gender, education level and the presence of a chronic illness. Several statistically significant differences were found (see Table 6). However, we believe that these patterns were likely due to real differences between subgroups, in support of the construct validity of the CADICEE tool, rather than indicative of potential differential item functioning. For example, it is understandable for patient advisors to report having more useful expertise during the consultation compared with patients in other clinical settings (*P* = .035) and for them to have a greater need for their expertise to be recognized and considered by the HCP (*P* < .001). In addition, patients with an elementary level of education generally required more help in becoming more autonomous compared with others, and patients with a university level had a lesser need in this regard (*P* = .037).

**TABLE 6:** Statistically significant differences in response patterns for CADICEE items among relevant respondent subgroups

Subgroups	Items with different response patterns* (difference**; *P* ***)
Clinical setting (*primary care vs specialty care vs patient advisors*)	‘Did you need your care to be adjusted to your personal context (family, work, values, etc)?’ (patient advisors > primary care ≈ specialty care; *P* = .016) ‘Did you need your expertise to be recognized and taken into account by professional?’ (patient advisors > primary care > specialty care; *P* < .001) ‘Did you receive important information about your health or care’ (specialty care < primary care ≈ patient advisors; *P* = .033) ‘Did you use your expertise in what was said or done’ (patient advisors > primary care > specialty care; *P* = .035)
Health‐care professional consulted (*doctor vs* *other*)	‘Did you feel that this professional recognized your expertise and took it into account?’ (doctor < other; *P* = .045)
Age (*18‐44 vs 45‐64 vs 65+*)	‘Did you feel that this professional had confidence in / trusted you?’ (46‐64 > 65+ > 18‐44; *P* = .037) ‘Did you receive important information about your health or care?’ (65+ > 45‐64 ≈ 18‐44; *P* = .017)
Gender (*female vs male*)	‘Did you need to feel empathy from this professional?’ (female > male; *P* = .021) ‘Did you need your expertise to be recognized and taken into account by professional?’ (female > male; *P* = .040) ‘Did you feel that this professional recognized your expertise and took it into account?’ (female > male; *P* = .036)
Education level (*elementary vs high school vs college/vocational vs university*)	‘Did you want this professional to help you become more autonomous / independent?’ (elementary > high school ≈ college/vocational > university; *P* = .037)
Presence of chronic illness (*yes vs no*)	‘Were you open to this professional's point of view (advice, etc)?’ (yes > no; *P* = .022)

* Median tests for independent medians performed on 18 out of the 24 CADICEE items. Six items with an overall median of 4 (maximum category) were excluded because all values among subgroups could only be below or equal to it.

** >: subgroup median *superior* to (…); <: subgroup median *inferior* to (…); ≈: subgroup median *equivalent* to (…).

*** Two‐tailed exact *P*‐values.

Finally, we computed the gaps between experience of care and needs, for every relevant pair of items in the CADICEE tool. For example, reporting that the care received was tailored to personal context only (2 = very little) when one had indicated a prior need for it to be tailored (4 = very much) would lead to a negative gap of 2 ranks (minus 2). We then created, for each respondent, an indicator representing the number of CADICEE dimensions exhibiting a ‘minor’ negative gap (minus 1 rank) and another indicator for ‘major’ negative gaps (minus 2 or 3 ranks). Respondents not having completed any pair of items in four CADICEE dimensions or more (n = 6) were excluded from this analysis. Based on these calculations, only 10% of participants did not experience any negative gap in partnership during their consultation. ‘Minor’ gaps were experienced by 85.4% of respondents on a median of 2 CADICEE dimensions (IQR = 2). ‘Major’ gaps were experienced by a third of participants (34.1%), with 4.8% experiencing a ‘major’ gap in four or more CADICEE dimensions. Associations between these indicators and respondents’ general appreciation of the consultation were both highly statistically significant and of increasing magnitude in the expected direction (*r*_s_ = −.24, *P* < .001 for ‘minor’ gaps; *r*_s_ = −.43, *P* < .001 for ‘major’ gaps), further supporting the tool's construct validity.

At the end of Step 4, the research team reviewed the usability and performance measurement results and submitted the questionnaire to the patient research advisory group one last time during a conference call and decided to add a final question to indicate the significance of each of the seven CADICEE dimensions during the consultation. Consequently, the final version, available in French and English, was adopted.^51^

## 3 DISCUSSION

### 3.1 Strengths of the tools

This CADICEE tool is, to our knowledge, the first developed in co‐construction with patients from development to validation ^19^, ^52^ integrating the GRIPP2‐LF recommendations.^32^ This co‐construction allowed us to call upon patients possessing a shared theoretical understanding of partnership and having developed competencies through their life experience with illness in relation to their HCPs. With the co‐construction, we have expanded our understanding by exploring how expert patients assess the quality of their partnership with their HCP, in a situated manner that is conditional on their current needs and expectations. Patients’ perspectives, and their on‐going participation in the development of tools or interventions intended for them, can therefore help ensure their appropriateness.^53^, ^54^, ^55^

The expert patients were able to transform the definition of partnership on seven dimensions^4^: **C**onfidence/trust, **A**utonomy, participation in **D**ecision‐making, sharing **I**nformation, personal **C**ontext, **E**mpathy and **E**xpertise. This tool provides a more granular, and still empirically grounded, definition of the concept of patients‐as‐partners in direct care, derived from what patients familiar with the concept consider significant when assessing changes in the quality of their partnership with HCPs.

This conception of partnership in care emerged far from its general definition in the literature, which places partnership practices at the highest level of the continuum of patient engagement.^3^, ^6^, ^55^, ^56^, ^57^ In fact, in previous works, partnership was seen as a level of co‐construction and co‐responsibility around clinical decision‐making. However, throughout the creation of the tool, patients stressed the importance of being primarily attentive to their needs and being able to find ways to discuss them so that professionals could respond to them. Furthermore, the partnership in care was defined as reflexive concomitant efforts to discuss, acknowledge and eventually increase the degree of congruence between patients’ needs in their interactions with their HCP and their perceptions of those interactions. Indeed, some patients are in situations where this is less suitable, or are not ready to be involved in their care. Professionals can then better support their patients by adjusting to their needs, and by encouraging a relationship in which the patient is increasingly involved. Therefore, this conceptualization of partnership has implications for HCPs’ practices, which are adapted to patients’ expectations as much as possible. This kind of measure can be used to improve HCPs’ practices on a regular or ad hoc basis, encouraging them to engage in a reflective/critical analysis of their interactions with patients.

### 3.2 Content validity and reliability

The CADICEE tool was designed and revised and involved numerous knowledgeable patients at every step, ensuring its content validity. The tool demonstrated high face validity, acceptability and relevance for both patients and HCPs. The 24 CADICEE items are easy to understand, and most patients should be able to complete them in under 20 minutes. We found evidence that the tool had sound construct validity and performed well in a wide range of clinical settings and with diverse adult patient populations. Further work could improve its usability in specific areas of care such as mental health. We believe the tool can be helpful to elicit gaps in care partnership and enhance relationships between HCPs and patients.

Future validation studies could look into the tool's test‐retest reliability, cross‐cultural validity and responsiveness to change. It was outside the scope of our work to assess whether patients’ assessments of CADICEE items would remain stable over time. Previous studies have reported acceptable temporal stability for several patient experience measures, especially for items rated on an ordinal scale such as the CADICEE items.^58^ Cross‐cultural validity could be especially important if the tool is to be used in jurisdictions where the concept of patients‐as‐partners is not yet well known and implemented in practice. We did not seek to assess criterion validity because there is no external gold standard for care partnerships, as is often the case in patient‐reported measures.^49^ However, the tool's construct validity could be further established by directly examining how CADICEE items relate to other validated scales pertaining to the same constructs (eg trust, shared decision‐making), when such scales exist.

### 3.3 Key features that make the tool unique

Some of the CADICEE dimensions such as confidence/trust, information‐sharing and empathy have been included in previous instruments measuring patient experience of care.^59^ However, it is worth emphasizing that the CADICEE tool approaches these dimensions from an innovative angle of reciprocal relationships between partners, acknowledging that patients are more than passive recipients of care, which is manifested in items measuring, for example, the professional's level of trust in the patient, the information shared by the patient with the professional and the empathy that the patient shows for the professional. Other dimensions of the tool focus on aspects of care measured only by more recent instruments, such as patient activation or empowerment^28^, ^60^, ^61^, ^62^ or shared decision‐making.^16^, ^63^, ^64^ Finally, the dimension related to recognizing and using patient and professional expertise in the consultation has, to our knowledge, never been addressed in other instruments and represents an innovation. CADICEE effectively considers all these dimensions in a single and relatively short tool, from the perspective of patients‐as‐partners in their care with specific needs in each consultation, which represents an original contribution.

A recent scoping review^19^ also emphasized that most of the tools evaluate dimensions related to one or two different concepts of patient engagement (patient‐centeredness, or empowerment or shared decision‐making), and a few tools simultaneously measure some dimensions related to all the different concepts of patient engagement. The CADICEE tool includes all patient partnership dimensions and was built in co‐construction with patients from development to validation, which was not previously developed in the literature.^19^

The tool can also be short due to general overarching statements rather than more numerous and specific items that could have referred more directly to some of their subcomponents or associated observable phenomena (eg behaviours), as recommended in psychometrics. The patient research advisory group was instrumental in ensuring that the definitions of dimensions remain sufficiently general and evocative to be easily understandable by patients. They preferred a tool that would provide a vaguely correct assessment of the partnership in care over one that is precise, but incorrect. Following a consultation, patients orient their conduct based on their subjective impressions, regardless of how these relate to a more objective or external assessment. This subjective perspective is what the CADICEE tool captures, and why it is intended as an instrument to promote further reflexive assessment, discussion and investigation by patients and HCPs.

### 3.4 Limitations of the tool

The tool development process revealed some limitations. First, the CADICEE tool was developed with patients‐as‐partners who were familiar with the partnership concept. Although on this subject they were clearly experts among patients, their perspectives on the important dimensions of partnership in care may not represent the views of all patients. In addition, participants involved in developing the tool were mainly women. However, patient participants were of all ages and with various chronic diseases, and as such, the dimensions of the tool were developed on the basis of a diversity of experiences. Although CADICEE is intended to be a generic tool to be used for various diseases (chronic or acute) and in different clinical settings, we have not developed it for use in a paediatric setting; a separate version of the tool would most likely need to be developed for that purpose.

## 4 CONCLUSION

To our knowledge, CADICEE is the first tool developed in co‐construction with patients that evaluates the degree of partnership in care as perceived by patients in their relationship with their HCPs, and that can be used in different clinical contexts (hospitalization/consultation; chronic/acute diseases). We encourage further applications of the tool in other Canadian provinces and countries and welcome their results to further refine the tool. An examination of the properties of the measurement scales will be presented in a future article. Moreover, we are currently developing a version for HCPs to measure the gap in perceptions between patients and professionals during the consultation.

## CONFLICT OF INTEREST

The authors declare that there is no conflict of interest.

## AUTHORS' CONTRIBUTIONS

MPP and NC conducted literature review. All authors were involved in the entire questionnaire development process, read and approved the final manuscript. MPP, NC, LN, CDG and DJG wrote the first version of the manuscript.

## Data Availability

This is an open access article under the terms of the Creative Commons Attributions Licence, which permits use, distribution and reproduction in any medium, provided the original work is properly cited
